# Kinetics and H_2_O Influence on NO*x* Trapping and Selective Catalytic Reduction over Ce/Pd Doping Catalyst

**DOI:** 10.3390/molecules29153457

**Published:** 2024-07-24

**Authors:** Li Yang, Tianshan Xue

**Affiliations:** 1 State Key Laboratory of Environmental Criteria and Risk Assessment, Chinese Research Academy of Environmental Science, Beijing 100012, China; yang.li@craes.org.cn; 2 Institute of Atmospheric Environment, Chinese Research Academy of Environmental Sciences, Beijing 100012, China

**Keywords:** diesel vehicle, oxygen vacancies, NO*x* storage reduction, kinetic model, water

## Abstract

In this paper, the removal effects and activation energy of Ce and Pd doping on pollutants (CO, C_3_H_6_, and NO) were comparatively analyzed by using characterization methods and constructed kinetic equations. Furthermore, the problems of the water influence mechanism on the NSR process were also discussed. The results show the following: (1) Pd doping effectively improves the removal of CO (80%) and C_3_H_6_ (71%) in the low-temperature section of the catalyst (150–250 °C) compared to Ce doping, while Ce doping exhibits excellent low-temperature conversion of NO. (2) The reaction activation energy of the LaKMnPdO_3_ catalyst was 9784 kJ/mol, which was significantly lower than that of the LaKMnCeO_3_ catalyst. (3) The presence of H_2_O has an important enhancement effect in the storage performance of the LaKMnPdO_3_ catalyst for NO*x* but decreases the catalytic reduction of NO. It provides a solution for the effective treatment of the increasing problems of particulate matter and ozone pollution.

## 1. Introduction

In recent years, vehicle emissions have become an important contributor to composite atmospheric pollution, resulting in haze and photochemical smog pollution. To date, although the atmospheric environment in China has exhibited continuous and rapid improvement, PM_2.5_ pollution has not been fundamentally controlled. Meanwhile, many cities across the country are facing challenges with the increased ozone concentrations, making it a significant factor affecting the air quality after PM_2.5_. NO_x_ emissions from motor vehicles can be rapidly converted into nitrates under unfavorable meteorological conditions with stability and high humidity, and nitrates are regarded as the largest and fastest-growing component of PM_2.5_ secondary particles, which are important precursors for ozone formation. Diesel-powered machinery contains high levels of NO_x_ and soot particles (the main source of PM_2.5_). However, soot particles are generated in high-temperature and oxygen-deficient conditions, while NO_x_ is formed in high-temperature and oxygen-rich conditions. Therefore, the technologies that reduce NO_x_ and soot particles in diesel vehicles have conflicting requirements [1], which limits the effectiveness of traditional three-way catalysts used in gasoline vehicles for purifying diesel exhaust emissions [2,3]. Additionally, the activity and regeneration capability of diesel exhaust catalysts are greatly affected by the lower exhaust temperature, higher oxygen content, and presence of water in diesel emissions. Therefore, it is of great practical significance to reveal the effect of water on the NO_x_ trapping efficiency of catalysts based on the characteristics of diesel-powered mechanical exhaust. 

Many researchers studied water resistance from catalyst material improvements, catalyst structure optimization, chemical mechanism, etc. The inhibitory effect of water vapor on the activity of precious metal catalysts at low temperatures can be reduced by optimizing the catalyst preparation process and structural design [4]. Weikun Ai. et al. improved the catalyst performance under water conditions through adjusting the interlayer spacing and surface chemistry. And they explored the intermediate products formed in the presence of H_2_O and their effects on catalytic activity using in situ characterization techniques [5]. Noble metal catalysts have attracted tremendous attention in NO_x_ storage reduction (NSR) because of their outstanding catalytic activity and stability. Zhou et al. investigated lanthanum cobalt-based perovskite catalysts before and after Pd doping, and based on the characterization analysis, it was found that the higher oxidation valence state of Pd after doping was favorable for the perovskite structure composition and the improvement of the resistance of the catalyst to high-temperature sintering. The resultant Pd-doped perovskite-based catalyst displayed excellent dispersion [6,7]. Due to the variable valence states, cerium-based oxides have become one of the most widely used rare earth oxides and, in particular, CeO_2_ exhibits excellent oxygen storage and oxygen release capabilities. When CeO_2_ is used as an active additive in catalytic materials, it can effectively enhance catalytic activity. A research team [8] prepared a Ce-doped modified catalyst through the urea method and revealed that the addition of Ce can inhibit the oxidation of NO to nitrate by surface oxygen, and thereby, the conversion rate of NO and the H_2_O resistance of the catalytic material were effectively enhanced.

In our previous study, it was found that K-doped perovskite catalysts exhibited high chemical stability, excellent thermal stability, and strong redox performance under high-temperature conditions and can modulate the valence state of Mn and the amount of active oxygen. Accordingly, the reaction between the catalyst and carbon soot particles can be promoted to realize the increased conversion rates of CO and C_3_H_6_, while a high level of NO conversion can be maintained at a relatively low temperature [9,10]. In this work, the effects of Pd and Ce on the morphology of LaKMnO_3_-type catalysts and the NO_x_ removal performance under oxygen-rich conditions will be investigated. The kinetic equation for the catalytic reduction of NO_x_ will be constructed. Moreover, water-containing experiments under lean and rich combustion conditions will be designed to further explore the impact mechanisms of water vapor on the NO_x_ storage and reduction process of catalysts.

## 2. Results and Discussion

### 2.1. Microstructure and Morphology

**Figure 1:**
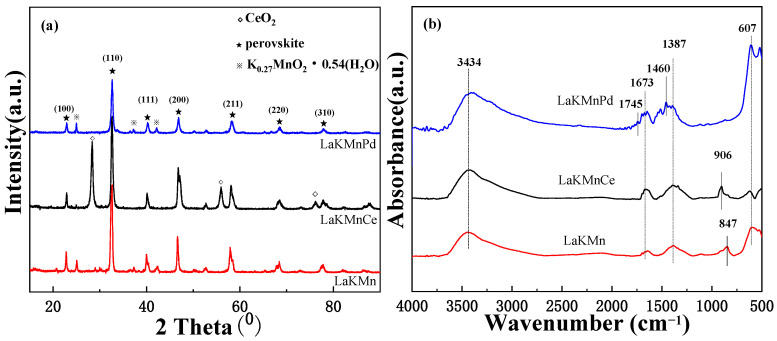
(**a**) X-ray diffraction patterns for catalysts; (**b**) FT-IR of LaKMnO_3_, LaKMnCeO_3_, and LaKMnPdO_3_ catalysts.

To investigate the crystal structure of the catalysts LaKMnO_3_, LaKMnCeO_3_, and LaKMnPdO_3_, the XRD patterns for the calcined samples are presented in Figure 1a. Three catalysts maintain a good characteristic peak of perovskite at 22° (100), 33° (110), 40° (111), 47° (200), 58° (211), 68° (220), and 77° (310), which are attributed to PDF#82-0232. It can be seen in Figure 1 that the intensities of characteristic diffraction peaks of perovskite are decreased with the doping of Ce and Pd, respectively. This can be explained by the fact that the defects are introduced into the structures of LaKMnCeO_3_ and LaKMnPdO_3_ after the doping of Ce and Pd, leading to the reduced intensities of characteristic diffraction peaks of perovskite. After Ce doping, the characteristic diffraction peaks of CeO_2_ can be detected, which is attributed to the larger ionic radius of Ce^3+^ compared to that of Mn^2+^. When Ce^3+^ is partially doped into the B site to replace Mn^2+^, the average ionic radius of B becomes larger to produce lattice distortion, causing the partial collapse of the perovskite structure and the formation of CeO_2_ [11]. Studies have demonstrated that the formation of CeO_2_ can be conducive to the generation of active oxygen, and thereby, the combustion activity of a catalyst towards soot particles can be improved. The catalyst doped with Pd shows a K_0.27_MnO_2_·0.54H_2_O (KMO) phase with the morphology of nanosheets, which is favorable for the rapid diffusion of electrons in the KMO structure, but no Pd-based phase can be detected. This may be attributed to the good incorporation of Pd into the crystal structure or the corresponding highly dispersed form on the catalyst surface.

Table 1 lists the lattice parameters and specific surface area of La_0.5_K_0.5_MnO_3_ doped with Ce and Pd. It can be seen that Ce doping leads to an increase in the lattice parameter *a* and a decrease in *b* and *c*, resulting in a reduction in grain size. This is because the ionic radius of Ce^3+^ (1.01 Å) is larger than that of Mn^2+^ (0.66 Å), causing an increase in the lattice parameter *a*. However, the electronegativity of Ce^3+^ (1.12) is smaller than that of Mn^2+^ (1.55), and the electrostatic repulsion between oxygen layers is reduced, so the interlayer spacing is decreased, leading to a reduction in the lattice parameter *c*. On the other hand, it can be found that Pd doping causes a decrease in the lattice parameter *a* and an increase in *b* and *c*, resulting in a reduction in grain size. This is mainly due to the fact that the ionic radius of Pd^2+^ (0.64 Å) is smaller than that of Mn^2+^, and thereby, the lattice parameter *a* is decreased. Nevertheless, the electronegativity of Pd^2+^ (2.2) is larger than that of Mn^2+^ (1.55), and thereby, the combination of Pd-O is prone to ionic bonds. This causes the electrostatic repulsion between oxygen layers to increase, and consequently, the interlayer spacing is enhanced, leading to an increase in the lattice parameter *c*. Furthermore, we find that Ce and Pd doping both increase the specific surface area of La_0.5_K_0.5_MnO_3_, which can provide a larger platform for catalytic reactions.

As shown in Figure 1b, all three catalysts, LaKMnO_3_, LaKMnCeO_3_, and LaKMnPdO_3_, have the stretching vibration peak of OH groups at 3424 cm^−1^ involved in hydrogen bonding, with the O-H bending peak at 1673 cm^−1^ being attributed to the hydroxyl group on the adsorbed water surface, and the characteristic vibration peak of free nitrate ions being at 1387 cm^−1^ [12,13]. The characteristic peak at 605 cm^−1^ is ascribed to the perovskite framework vibration. The peaks at 906 cm^−1^ and 848 cm^−1^ are assigned to carbonate vibrations. After Pd doping, the LaKMnPdO_3_ catalyst shows the characteristic peaks of K_2_CO_3_ at 1745 cm^−1^ and 1460 cm^−1^. The formation of carbonates makes contributions to the reaction between the catalyst and NO_x_ to generate nitrates, thereby improving the conversion rate of NO and enhancing the activity of the catalyst [14]. Compared with the LaKMnO_3_ catalyst, the carbonate absorption peaks of the LaKMnCeO_3_ and LaKMnPdO_3_ catalysts around 1387 cm^−1^ and 906 cm^−1^ are significantly larger in peak width and higher in peak intensity. This indicates that the doping of Ce and Pd can improve the adsorption of NO by the catalyst, further leading to an increase in the conversion rate of NO.

**Figure 2:**
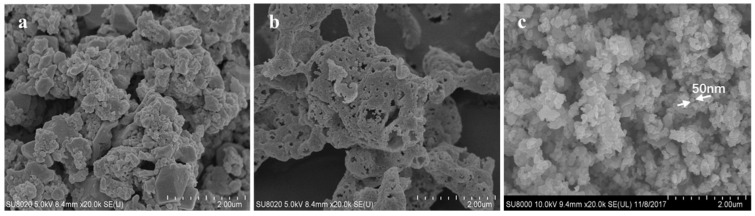
SEM images of catalysts: (**a**) LaKMnO_3_; (**b**) LaKMnCeO_3_; (**c**) LaKMnPdO_3_.

In the catalytic reaction involving gas–solid contact, the surface structure of the catalyst plays a significant role in catalytic activity. In order to further analyze the influences of the structure of a catalyst surface on the catalytic activity, as shown in Figure 2, SEM observations at the magnification of ×20,000 were performed on the three catalysts, including LaKMnO_3_, LaKMnCeO_3_, and LaKMnPdO_3_. It is apparent that the LaKMnO_3_ particles are irregular in size and poorly dispersed, whereas the catalyst particles doped with Ce or Pd exhibit more uniform dispersion and a spherical shape with the particle size range of 50–100 nm, and they are arranged more loosely. This is consistent with the results in Table 1.

### 2.2. Catalytic Activity Tests

**Figure 3:**
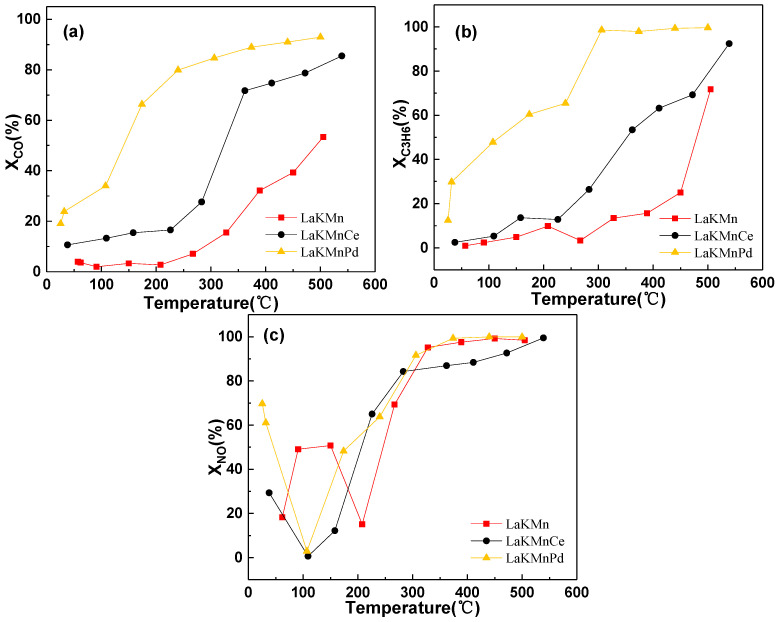
C_3_H_6_, CO, and NO conversion for catalysts. (**a**) CO conversion; (**b**) C_3_H_6_ conversion; (**c**) NO conversion.

**Figure 4:**
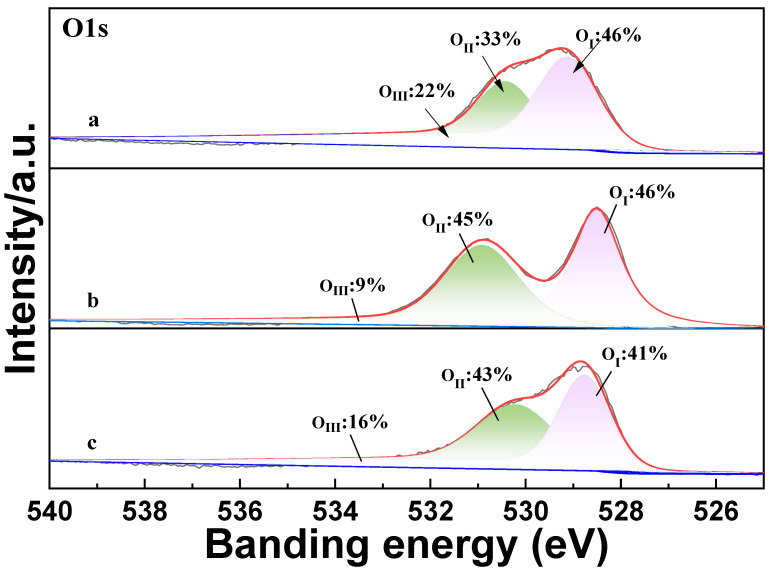
XPS of catalysts ((**a**) LaKMnO_3_, (**b**) LaKMnCeO_3_, and (**c**) LaKMnPdO_3_).

Figure 3 shows the conversion rates of C_3_H_6_, CO, and NO for the LaKMnO_3_, LaKMnCeO_3_, and LaKMnPdO_3_ catalysts. As shown Figure 4a, the conversion rate of C_3_H_6_ is enhanced with the increase in temperature. After Pd doping, the LaKMnPdO_3_ catalyst exhibits the best performance in removing C_3_H_6_, reaching a conversion rate above 90% at 288 °C. In contrast, after Ce doping, the LaKMnCeO_3_ catalyst reaches a conversion rate above 90% only at a temperature of 530 °C. As shown in Figure 4b, the CO conversion rates of the three catalysts are also gradually improved with the increase in temperature. The Pd-doped catalyst shows the best performance in CO removal, reaching a conversion rate of 50% at 141 °C. In contrast, the Ce-doped catalyst reaches a CO conversion rate of 50% at 324 °C, while the undoped LaKMnO_3_ catalyst exhibits the lowest CO conversion rate of 50% at 492 °C.

For the NO conversion rates, as shown in Figure 3c, all three catalysts exhibit certain storage properties for NO. The NO conversion rate curves for all of the catalysts begin with a relatively high conversion rate, and then they reach a minimum conversion rate point after increasing the temperature. Both LaKMnCeO_3_ and LaKMnPdO_3_ reach nearly zero conversion rates at their minimum points. This is because during the low-temperature stage, the oxidation of NO and the adsorption reaction of NO_x_ mainly occur. The oxidized and adsorbed NO_x_ is stored on the K sites. After reaching a certain temperature, the conversion rates approach zero, indicating that the emitted NO concentration is close to the intake NO concentration, and thus, the adsorption reaches saturation. The conversion rates of LaKMnCeO_3_ and LaKMnPdO_3_ approach zero at 109 °C and 107 °C, respectively, while the undoped LaKMnO_3_ reaches its lowest conversion rate of NO at 207 °C. This indicates that the NO storage capability of a catalyst can be enhanced by Ce/Pd doping, so NO adsorption and oxidation occur at a relatively low temperature. Meanwhile, as the temperature further increases, the conversion rate of NO is gradually increased. However, due to the thermodynamic equilibrium limitations on the oxidation of NO at high temperatures [15], the increase in the NO conversion rate during the high-temperature stage is mainly attributed to the desorption and decomposition of the NO_x_ stored in the catalyst. By comparing the NO conversion rates of Ce- and Pd-doped catalysts, it can be found that Ce-doped LaKMnCeO_3_ exhibits a NO conversion rate of 74% at a low temperature (250 °C), which is higher than that of LaKMnPdO_3_ (68%).

**Figure 5:**
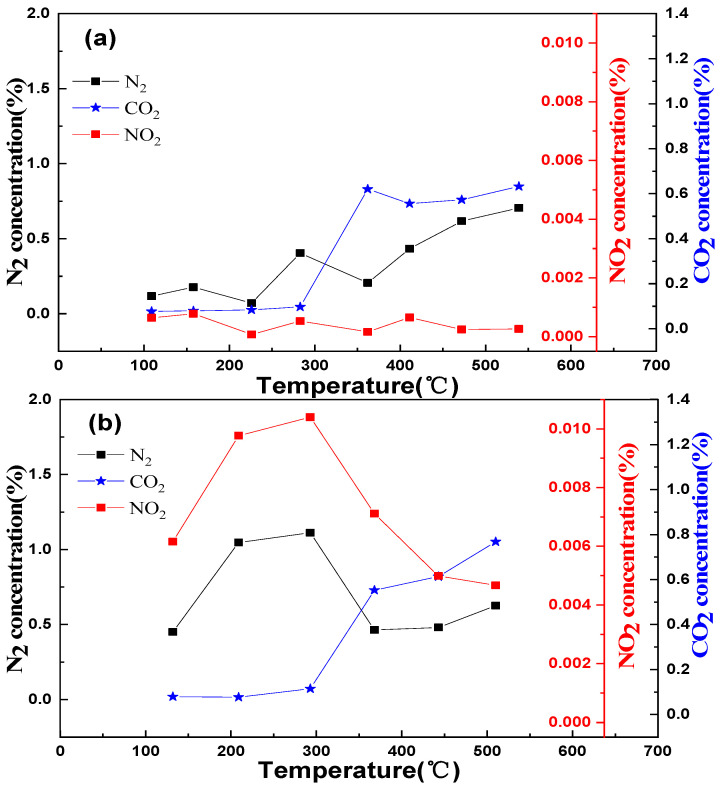
Results of De-NO_x_ process performed for catalysts ((**a**) LaKMnCeO_3_; (**b**) LaKMnPdO_3_).

The obtained samples were tested in the role of the two catalysts (LaKMnCeO_3_ and LaKMnPdO_3_) of NO_x_ storage and reduction. Figure 5 shows the results of the De-NO_x_ process, which further confirms the reaction process in Figure 3.

There are three forms of oxygen in the XPS peak spectrum of O1s, which are lattice oxygen O_I_ in the range of 528.5–530.2 eV and the intermediate binding energy peak O_II_ at 531–532 eV, assigned to the adsorbed oxygen, and high-binding oxygen O_III_ around 533 eV corresponding to the hydroxyl group on the surface [16,17]. Figure 4 reveals that after K and Ce co-doping, the ratio of the peak area of the high-binding O_II_ and low-binding energy peak O_I_ of the catalyst increased significantly. To clarify the content changes in the different forms of oxygen in the catalyst, we performed a semiquantitative analysis of the O1s spectrum. The result shows that Ce and Pd doping increases the content of adsorbed oxygen, which enhances the oxidation activity of the catalyst. Additionally, the quantitative results illustrate that Pd doping increases the O_III_ content, which contributes to improving the catalyst’s selectivity and NO_x_ conversion rate.

Figure 5 shows the reaction process of the LaKMnCeO_3_ and LaKMnPdO_3_ catalysts under oxygen-rich conditions. It is obvious that the concentrations of CO_2_ in the catalysts doped with Ce and Pd are gradually improved with the increasing temperature. With the low temperature range (100–400 °C), the concentrations of N_2_ and NO_2_ for catalyst b (LaKMnPdO_3_) are remarkably higher than that of catalyst a (LaKMnCeO_3_). This suggests that more oxygen vacancies can be generated by Pd doping, which enhances the mobility of oxygen and effectively improves the NO_x_ storage capacity of a catalyst at a low temperature. After 400 °C is reached for the LaKMnCeO_3_ and LaKMnPdO_3_ catalysts, the concentrations of CO_2_ are gradually increased, whereas the concentrations of NO_2_ are decreased. This is because the stored NO_x_ experiences desorption and decomposition and then reacts with reducing gasses such as CO and HC, leading to an increase in the concentration of CO_2_. It should be noted that this is consistent with the results of the conversion rate curves in Figure 3.

### 2.3. Kinetics of LaKMnCeO_3_/LaKMnPdO_3_ Catalyst on NO_x_ Removal

According to the law of mass action, it can be determined that in a typical elementary reaction, the reaction rate is directly proportional to the product of the absolute values of the stoichiometric coefficients and the concentrations of the reactants raised to the power of their respective coefficients in the reaction equation. Nevertheless, for non-elementary reactions, the reaction rate cannot follow the law of mass action, and its reaction order is not necessarily equal to the stoichiometric coefficients of the reactants in the reaction equation. In order to deeply understand the catalytic oxidation of NO by the LaKMnCeO_3_/LaKMnPdO_3_ catalyst, we firstly need to determine the reaction order of this reaction. Furthermore, the chemical reaction rate is also closely related to the magnitude of the reaction activation energy. It should be mentioned that a lower activation energy can result in a faster reaction rate, so the reduced activation energy can effectively promote the progress of the reaction.

#### 2.3.1. Reaction Order of LaKMnCeO_3_

According to the kinetic parameter experiments conducted on LaKMnCeO_3_, the conversion rate (α) of NO and the reaction rate (r_NO_) of NO can be obtained as listed in Table S2. The logarithms of the NO concentration (lgC_NO_) and NO reaction rate (lgr_NO_) were taken, and then linear fitting was performed with lgC_NO_ as the *x*-axis and lgr_NO_ as the y-axis. As presented in Figure 4 (left), the linear fitting equation can be determined as y = −3.2426x + 12.146 with a correlation coefficient of R^2^ = 0.7702. The resultant slope of the equation corresponds to the reaction order of the NO concentration in the catalytic reaction of LaKMnCeO_3_ in which a = −3.2426.

**Figure 6:**
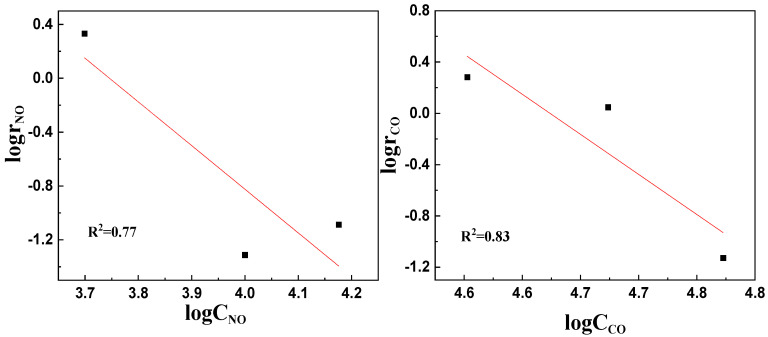
Fitting curves of lgr_NO_, lgC_NO_ and lgr_CO_, lgC_CO_.

Based on the CO conversion rate (α) and CO reaction rate (r_NO_) in Table S2, the logarithms of the CO concentration (lgC_CO_) and CO reaction rate (lgr_NO_) were taken, and then linear fitting was performed with lgC_CO_ as the *x*-axis and lgr_NO_ as the *y*-axis. As illustrated in Figure 6 (right), the linear fitting equation can be determined as y = −7.8086x + 36.38 with a correlation coefficient of R^2^ = 0.8296. The resultant slope of the equation corresponds to the reaction order of the CO concentration in the catalytic reaction of LaKMnCeO_3_ in which b = −7.8086.

For the temperature gradient experiment, the concentrations of NO and CO were set as constants with a total gas flow rate of 500 mL/min, and the catalyst mass was 0.45 mg. Accordingly, the conversion rate (α) and NO reaction rate (r_NO_) are shown in Table S3. Linear fitting was performed with RT/1 as the *x*-axis and lgr_NO_ as the *y*-axis. The fitting equation was obtained as y = 14,916x + 1.3694 with a correlation coefficient of R^2^ = 0.7084. According to the Arrhenius equation, both sides of Equation (10) were taken by logarithm, and Equation (1) can be obtained. Therefore, the slope of the fitting equation corresponds to the activation energy of the reaction, Ea = 14,916 kJ/mol, while the intercept of the equation is lnA + lnf(x), so the pre-exponential factor A can be achieved.

(1) $$lnr_{NO}=\left[lnA+lnf\left(x\right)\right]-\frac{Ea}{RT}$$

Eventually, the kinetic equation for the catalytic oxidation of NO by LaKMnCeO_3_ can be determined as follows:

(2) $$r=0.74e^{\frac{14280}{RT}}{\left[\mathrm{NO}\right]}^{-3.2436}{\left[\mathrm{CO}\right]}^{-7.8086}$$

#### 2.3.2. Reaction Order of LaKMnPdO_3_

**Figure 7:**
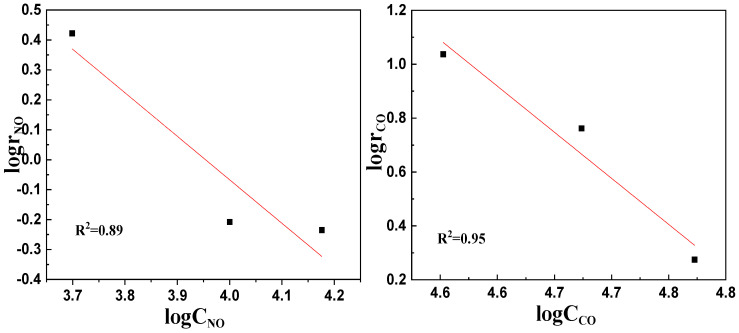
Fitting curves of lgr_NO_, lgC_NO_ and lgr_CO_, lgC_CO_.

Similarly, the NO conversion rate (α) and NO reaction rate (r_NO_) could be obtained from the kinetic experiment of the LaKMnPdO_3_ catalyst, and then the corresponding logarithms were taken to achieve the lgr_NO_ and lgC_NO_ values, as shown in Figure 7 (left). Linear fitting was performed with lgC_NO_ as the *x*-axis and lgr_NO_ as the *y*-axis. The linear fitting equation can be determined as y = −1.454x + 5.7491 with a correlation coefficient of R^2^ = 0.8904. The slope of the equation corresponds to the reaction order of the NO concentration in the catalytic reaction of LaKMnPdO_3_ in which a = −1.454.

The logarithms of the CO concentration and CO reaction rate were taken as lgr_CO_ and lgC_CO_, and linear fitting was performed with lgC_CO_ as the *x*-axis and lgr_NO_ as the *y*-axis, as shown in Figure 7 (right). The linear regression equation about the catalytic reaction rate of LaKMnPdO_3_ can be determined as y = −4.2761x + 20.76 with a correlation coefficient of R^2^ = 0.9537. The slope of the equation corresponds to the reaction order of the CO concentration in the catalytic reaction of LaKMnPdO_3_ in which b = −4.2761.

The temperature gradient experiment parameters for LaKMnPdO_3_ were the same as those for LaKMnCeO_3_. Based on the temperature gradient experiment, the conversion rate (α) and NO reaction rate (r_NO_) can be obtained, as shown in Table S3. Similarly, linear fitting was performed with RT/1 as the *x*-axis and lgr_NO_ as the *y*-axis. The fitting equation can be obtained as y = 9784.4x + 1.0041 with a correlation coefficient of R^2^ = 0.7909. The activation energy of the reaction can be obtained as Ea = 9784.4 kJ/mol, and the pre-exponential factor, A, can be determined as 1.23. Eventually, the kinetic equation for the catalytic oxidation of NO by LaKMnPdO_3_ can be achieved as follows:

(3) $$r=1.23\cdot e^{-\frac{9784.4}{RT}}{\left[\mathrm{NO}\right]}^{-1.454}{\left[\mathrm{CO}\right]}^{-4.2761}$$

According to the kinetic reaction Equations (2) and (3) of LaKMnPdO_3_ and LaKMnCeO_3_ obtained by linear fitting, it can be found that the activation energy, Ea, for LaKMnPdO_3_ (9784.4 kJ/mol) is remarkably lower than that for LaKMnCeO_3_ (14,916 kJ/mol). Therefore, the LaMnO_3_-based catalyst doped with Pd exhibits better efficiency in NO removal compared to its Ce-doped counterpart.

### 2.4. Water Effects on LaKMnPdO_3_ Activity

In order to further understand the performance changes in the LaKMnPdO_3_ catalyst with a superior NO_x_ storage capacity under water-containing conditions, property tests were carried out under different water flow conditions.

**Figure 8:**
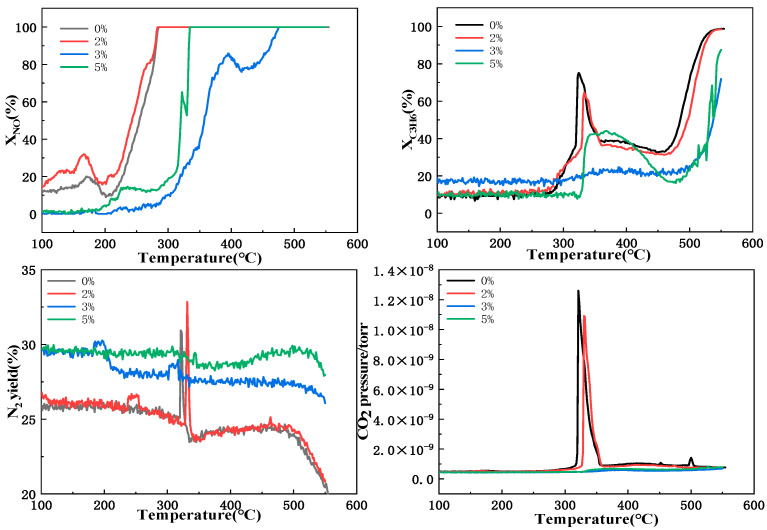
Catalytic performance of LaKMnPdO_3_ for reduction of C_3_H_6_, NO, and N_2_ yielsd under different water volumes.

Figure 8 shows the catalytic performance of LaKMnPdO_3_ for the reduction in the C_3_H_6_, NO, and N_2_ yields under four different water volumes (0%, 2%, 3%, and 5%). For the variations in the conversion rate of NO, as shown in Figure 8, the addition of an appropriate amount of water (2%) is conducive to improve the conversion rate of NO by the catalyst at low temperatures (150–200 °C). The highest conversion rate at a low temperature of 166 °C reaches 33% for the catalyst with 2% water flow, whereas the catalyst without water flow exhibits a maximum conversion rate of 20% at a low temperature. The negative peaks in the conversion rate of NO appear at 329 °C and 416 °C for the water flow rates of 3% and 5%, respectively. This is probably attributed to the competition between excessive water and NO_x_ for adsorption on the potassium sites, leading to the formation of KOH. However, KOH on the surface is unstable and decomposes at a temperature around 380 °C [18,19], and thus, as the water flow rates are 3% and 5%, NO decomposes, desorbs, and migrates to the surface at around 329 °C and 416 °C, respectively. For the conversion efficiency diagram of C_3_H_6_, it can be found that the addition of an appropriate amount of water cannot greatly affect the conversion rate of C_3_H_6_, while excessive water flow leads to a significant decrease in the conversion rate of C_3_H_6_. Nevertheless, there is a significant increased peak at 343 °C at a water flow rate of 5% compared to that obtained at a 3% water flow rate. This phenomenon may be due to the presence of excessive water promoting the direct reaction of NO_x_ with water to generate HNO_2_ or HNO_3_ or directly reacting with the surface soot and hydrocarbons through a reforming reaction [20,21], instead enhancing the oxidation of hydrocarbons. For the N_2_ yield diagram, it can be found that the addition of an appropriate amount of water can enhance the conversion rate of NO_x_ → N_2_. The N_2_ yield increases from 31% without water flow to 33% with a water flow of 2%. This may be due to the presence of an appropriate amount of water, which can promote the surface diffusion rate of nitrogen species, but excessive water flow can block the active reaction sites of the catalyst], thus reducing the catalytic reduction performance of NO.

**Figure 9:**
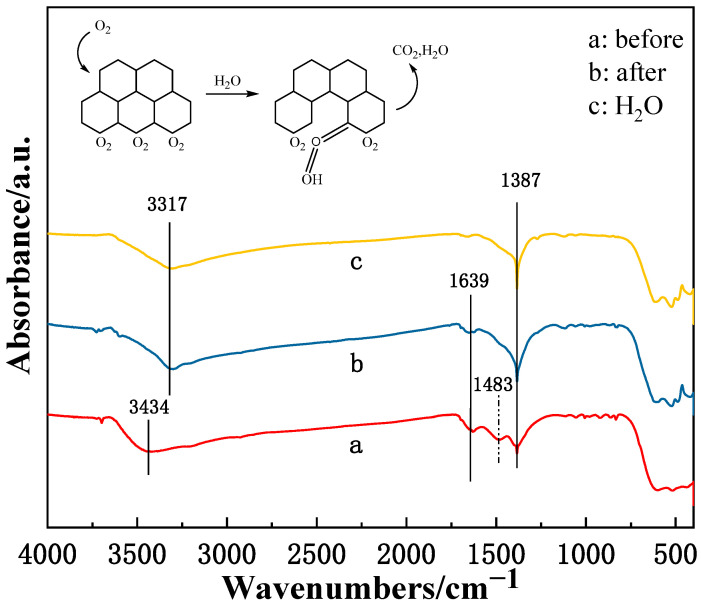
The FT-IR of LaKMnPdO_3_ with different active component loadings: (**a**) before the experiment; (**b**) after the experiment; and (**c**) in the presence of water.

Figure 9 shows the FTIR results of the LaKMnPdO_3_ catalyst before and after the anhydrous activity test and after the water activity test. The stretching vibration peak of -OH appears at 3446 cm^−1^, and the stretching vibration peak of hydrogen-bonded OH appears at 3317 cm^−1^. Compared with catalyst a, the shape of the hydroxyl stretching vibration peak in the catalyst system of catalyst b remains essentially unchanged, but the peak intensity increases, and the peak position shifts to a lower wavenumber (red shift), which indicates that the hydrogen bonding mode of hydroxyl groups in the system has changed. The peak at 1639 cm^−1^ is attributed to the O-H bending vibration of the adsorbed water surface hydroxyl groups. The peak at 1387 cm^−1^ is characteristic of free nitrate ions. The peak at 1483 cm^−1^ is attributed to the characteristic absorption of K_2_CO_3_ while no distinct K_2_CO_3_ phase was detected in the XRD, indicating that K_2_CO_3_ is dispersed on the surface of the catalyst support. In Figure 8, it can be seen that after the temperature-programmed experiment, the characteristic peak of nitrates (1381 cm^−1^) gradually replaced the characteristic peak of carbonates (1483 cm^−1^). This indicates that carbonates contribute to the storage of NO_x_. Meanwhile, the O-H bending vibration peak of the catalyst disappears after water exposure, and the nitrate characteristic peak becomes sharper, which illustrates that water addition changes the O_2_ activation mechanism and promotes nitrate formation. Based on the above analysis results, we deduce the mechanism of water effects on the catalytic soot combustion process, as illustrated in Figure 8. First, gas-phase oxygen adsorbs onto the free carbon sites on the soot surface. Then, with the addition of water, hydrogen bond interactions between water molecules and oxygen lead to the formation of O_2_…H_2_O complexes. Second, the hydrogen atoms from the water transfer to the adsorbed O_2_ and form OOH groups in the presence of soot. And as the temperature rises, these OOH groups react with soot over the catalyst, producing CO_2_ and H_2_O.

**Figure 10:**
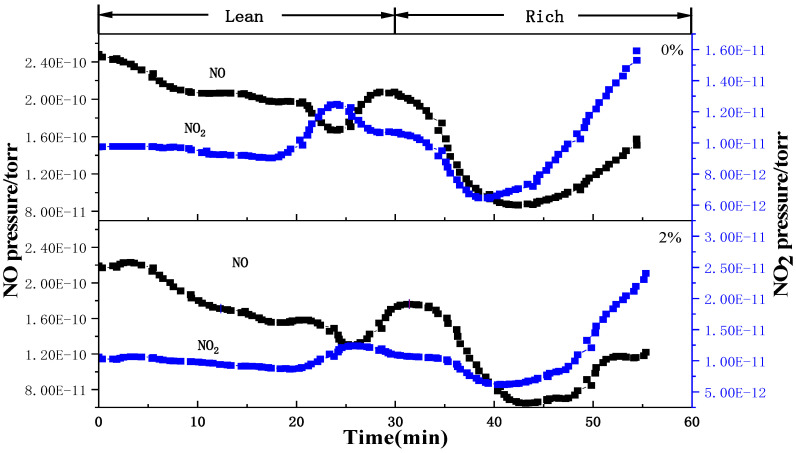
Alternate experimental results of rich and lean combustion of LaKMnPdO_3_ catalyst under water-free and water conditions.

Figure 10 presents the alternate experimental results of the rich and lean combustion of the LaKMnPdO_3_ catalyst under water-free and water conditions. Based on the conversion rate curves of NO and NO_2_, it can be seen that the NO_x_ storage performance of the catalyst can be enhanced by the addition of a small amount of water under a lean combustion condition, and the equilibrium time of NO_x_ storage is extended from 17 min to 22 min, but the amount of NO_2_ cannot be correspondingly increased. It suggests that NO cannot be mainly converted into NO_2_ for storage after water is added, but it is converted into other forms to promote the increase in the NO conversion rate. This may be due to the competition between water and NO_x_ for adsorption on the alkaline sites, and thereby, the hydroxyl compounds are generated, which react with oxygen and NO_x_ to form nitrates, promoting the conversion of NO [22,23]. However, during the rich combustion stage (30–60 min), it can be found that the desorption amount of NO_x_ under water flow conditions is significantly lower than that under water-free conditions. This indicates that the hydroxyl compounds generated during the lean combustion stage interact with the stored NO_x_ to form more stable nitrates, which are not easy to decompose, thereby affecting the desorption of NO_x_ and causing a decrease in the NO conversion rate, as shown in reactions (4) and (5).

K_2_O + H_2_O → 2KOH (4)

2KOH + 2NO + O_2_ → 2KNO_2_ + H_2_O (5)

## 3. Materials and Methods

### 3.1. Catalyst Synthesis

The La_0.5_K_0.5_MnO_3_^−^-supported Ce/Pd were prepared through the citrate complex method. According to the stoichiometric ratio, LaNO_3_, KNO_3_, Ce(NO_3_)_2_·6H_2_O/PdCl_2_, and Mn(NO_3_)_2_·6H_2_O are separately dissolved in an appropriate amount of deionized water. The citric acid was dissolved in an appropriate amount of deionized water based on the molar ratio of the total citric acid/metal ions, which is 1:1.2, and then it was mixed with the metal nitrate solution, which was prepared in the first step to obtain a wet gel [24]. The obtained wet gel was dried at a constant temperature of 120 °C for 12 h and calcined under air flow in a muffle furnace at 400 °C for 2 h, and then it was warmed up to 700 °C for 4 h. Finally, the calcined black powder was crushed to a 400 mesh size and placed in a drying dish for reserve.

### 3.2. Catalyst Characterization and Kinetics Analysis

X-ray diffraction (XRD) patterns were used to determine the phases of catalysts. All catalysts were measured in the 2θ range from 10° to 90° with a diffraction meter using Cu Kα radiation, generator power values of 40 kV and 40 mA, a step size of 0.02°, and a speed of 6°/min. A scanning electron microscopy (SEM; Hitachi, Japan) with an accelerating voltage of 15 kV and 1000–50,000 amplification factor was used to analyze the morphology of the catalyst. The surface functional groups and valence bonds of the catalyst were verified by Fourier transform infrared spectroscopy (FTIR; Magna-IR750, Nicolet, QC, Canada), which has a wavenumber range of 4000–450 cm^−1^.

The TPR experiment was carried out in a self-designed tubular fixed-bed reactor. Temperature-programmed reaction was conducted with a gas hourly space velocity (GHSV) of 16,000 h^−1^. The catalysts were directly exposed to reaction gas containing NO (0.1%), C_3_H_6_ (0.05%), CO (0.5%), and O_2_ (10%). The composition of the gas mixture produced from the reaction was analyzed using an online A5000 model gas chromatograph. Table S1 lists the experimental flue gas parameters.

In this paper, the catalytic activity for the investigated catalysts was evaluated by CO conversion (X_CO_), NO conversion (X_NO_), and soot initiation temperature (T_i_). In the reaction process, the conversion rate of the reaction gas was calculated using the following formula, and the soot initiation temperature is the temperature at which the tangent is intersected with the zero concentration at the slope of the maximum peak of the CO_2_ concentration rate change. T_i_ is calculated from a differential drawing of the CO_2_ concentration curve by making a tangent line at the slope of the maximum peak of the concentration rate change to the temperature where it intersects with zero concentration.

(6) $$X_{NO}=\frac{{\left[NO\right]}_{i}-{\left[NO\right]}_{o}}{{\left[NO\right]}_{i}}\times 100\%$$

(7) $$X_{CO}=\frac{{\left[CO\right]}_{i}-{\left[CO\right]}_{o}}{{\left[CO\right]}_{i}}\times 100\%$$

where *X*: conversion rate; *i*: inlet concentration; and *o*: export concentration.

In the kinetic reaction, pollutants are adsorbed on the catalyst surface and then diffused from the catalyst surface into the internal pore structure of the catalyst, where chemical reactions occur between gasses colliding on the catalyst surface as the temperature changes. In the LaKMnCeO_3_/LaKMnPdO_3_ kinetic parameter experiments, the catalyst mass was 0.45 g, the total gas flow rate was 500 mL/min, and the temperature was set at a constant 350 °C, while the NO and CO concentrations are different (Table S2). When different temperature (250–450 °C) gradient experiments were performed (Table S3), the NO and CO concentrations were constant at 2.5 mL and 25 mL, respectively.

The catalytic reaction rate equation for NO and CO in this study in the presence of a catalyst is shown below:

(8) $$r_{NO}=kC_{NO}^{a}C_{CO}^{b}$$

where $r_{NO}$: reaction rate in mol/g/s; *k*: reaction constant; a: reaction level corresponding to NO concentration; b: reaction level corresponding to CO concentration; C_NO_: NO volume concentration, ppm; and C_CO_: reaction level corresponding to CO concentration, ppm.

The reaction rate $r_{NO}$ can be calculated from the experimentally measured NO conversion, NO inlet concentration, total gas flow rate, and catalyst mass as follows:

(9) $$r_{NO}=\frac{\alpha \cdot C_{NO}\cdot L/60}{22.4M}$$

where *α*: conversion rate, %; *L*: total gas flow, mL; and *M*: catalyst mass, g.

The reaction rate of $r_{NO}$ can be calculated from the Arrhenius equation and combined with (8) and (9):

(10) $$r_{NO}=A\cdot e^{-Ea/RT}\cdot f(x)$$

(11) $$f(x)=C_{NO}^{a}C_{CO}^{b}$$

## 4. Conclusions

In this work, LaKMnCeO_3_ and LaKMnPdO_3_ catalysts were prepared by the citrate complex method. The effects of Ce/Pd doping on the NO_x_ storage and reduction performance of the catalysts were systematically investigated, and a kinetic model was established. It was found that the NO_x_ storage performance of the catalyst was significantly improved by Ce/Pd doping. Moreover, within a low temperature range, more oxygen vacancies can be generated by Pd doping to enhance oxygen mobility, which can effectively improve the NO_x_ storage performance of catalysts at a low temperature. Meanwhile, a kinetic analysis demonstrated that the activation energy of the LaKMnPdO_3_ catalyst was lower than that of the LaKMnCeO_3_ catalyst, which is beneficial to the occurrence of NO storage and reduction reaction, resulting in superior NO removal performance of the Pd-doped LaMnO_3_-type catalyst.

The introduction of an appropriate amount of water can prolong the equilibrium time of the catalyst for NO_x_ storage, leading to an enhanced NO_x_ storage performance. During the lean combustion stage, NO_x_ was stored in the form of nitrates on the chemical sites to promote the conversion of NO. However, during the rich combustion stage, the presence of water led to the formation of more stable nitrates, and thereby, the desorption of NO_x_ was inhibited, causing a decrease in the conversion rate of NO.

## Figures and Tables

**Table 1:** Lattice parameters of catalysts.

Composition	Lattice Parameters (Å)			Crystallite Size (nm)	Surface Area (BET)
	a	b	c		
La_0.5_K_0.5_MnO_3_	14.39	12.58	15.74	20.0	4.2
La_0.5_K_0.5_Mn_0.7_Ce_0.3_O_3_	20.05	8.36	15.43	18.1	17.49
La_0.5_K_0.5_Mn_0.97_Pd_0.03_O_3_	9.53	14.28	16.35	18.3	10.5

## Data Availability

The original contributions presented in the study are included in the article/Supplementary Materials, further inquiries can be directed to the corresponding author.

## Supplementary Materials

The following supporting information can be downloaded at https://www.mdpi.com/article/10.3390/molecules29153457/s1, Table S1: Experimental flue gas parameters. Table S2: The effect of the NO concentration. Table S3: Experiment of temperature effect.
