# Supplementary material for: Kinetics and H2O Influence on NOx Trapping and Selective Catalytic Reduction over Ce/Pd Doping Catalyst
Source: Molecules. 2024 Jul 24;29(15):3457. doi: 10.3390/molecules29153457 (PMC11313542; doi:10.3390/molecules29153457)
Supplement: Supplementary file 1 [file molecules-29-03457-s001.zip › molecules-3021695-supplementary.pdf]

Supporting Information

Kinetics and H_2O Influence on NO_x Trapping and Selective Catalytic Reduction over Ce/Pd Doping Catalyst

Li Yang [1,2] and Tianshan Xue [1,2,*]

1 State Key Laboratory of Environmental Criteria and Risk Assessment, Chinese Research Academy of Environmental Science, Beijing 100012, China; yang.li@craes.org.cn
2 Institute of Atmospheric Environment, Chinese Research Academy of Environmental Sciences, Beijing 100012, China
* Correspondence: lynnleslie@foxmail.com

The TPR experiment was carried out in a self-designed tubular fixed-bed reactor. The temperature-programmed reaction was conducted with a gas hourly space velocity (GHSV) of 16000 h-1. The catalysts were directly exposed to reaction gas containing NO (0.1%), C_3H_6 (0.05%), CO (0.5%), and O_2 (10%). The composition of the gas mixture produced from the reaction was analyzed using an online A5000 model gas chromatograph.

Table S1. Experimental flue gas parameters.

Gas	SO_2 (ppm)	NO (mL)	CO (mL)	C_3H_6 (mL)	O_2 (mL)	Ar (mL)	Total flow (mL/min)
Flow	300	0.25	1.25	0.125	25	208.7	250

The catalytic reaction rate equation for NO and CO in this study in the presence of a catalyst is shown below:

$$r_{NO} = kC_{NO}^{a}C_{CO}^{b} \quad \text{(S1)}$$

where r_{NO}: reaction rate, mol/g/s; k: reaction constant; a: reaction level corresponding to NO concentration; b: reaction level corresponding to CO concentration; C_{NO}: NO volume concentration, ppm; and C_{CO}: reaction level corresponding to CO concentration, ppm.

Table S2. The effect of the NO concentration.

Catalyst	NO concentration (ppm)	NO conversion rate (α)	NO reaction rate	CO concentration (ppm)	CO conversion rate (α)	CO reaction rate
$LaKMnPdO_3$	5000	0.640	2.645	40000	0.330	10.913
	10000	0.075	0.620	50000	0.140	5.787
	15000	0.047	0.583	60000	0.038	1.885
$LaKMnCeO_3$	5000	0.520	2.149	40000	0.058	1.918
	10000	0.0059	0.049	50000	0.027	1.116
	15000	0.0066	0.082	60000	0.0015	0.074

Table S3. Experiment of temperature effect.

Catalyst	Temperature (^{0}C)	Conversion rate (α)	Reaction rate
$LaKMnPdO_3$	250	0.230	0.048
	350	0.690	0.200
	450	0.500	0.186
$LaKMnCeO_3$	250	0.030	0.006
	350	0.24	0.069
	450	0.13	0.048
